# Supplementary material for: Genomic characterization of genes encoding histone acetylation modulator proteins identifies therapeutic targets for cancer treatment
Source: Nat Commun. 2019 Feb 13;10:733. doi: 10.1038/s41467-019-08554-x (PMC6374416; doi:10.1038/s41467-019-08554-x)
Supplement: Supplementary file 1 — Supplementary Information [file 41467_2019_8554_MOESM1_ESM.pdf]

Supplementary Material

Genomic characterization of genes encoding histone acetylation modulator proteins identifies therapeutic targets for cancer treatment

Zhongyi Hu et al.

Supplementary Figures

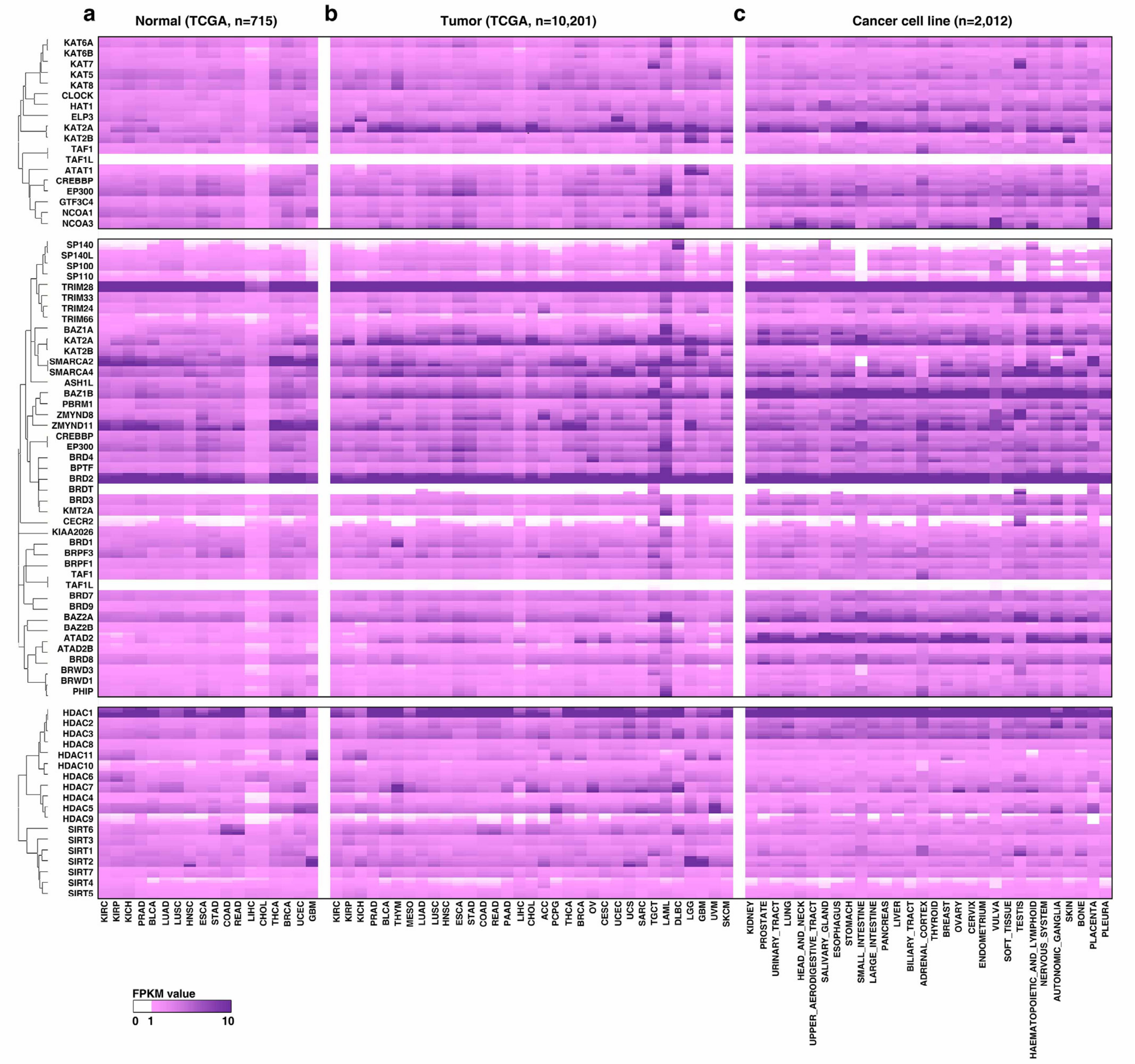

Supplementary Figure 1. Ubiquitous expression of the HAMP genes across normal tissues and cancers

a Heatmap shows the mRNA expression levels of the HAMP genes across corresponding adjacent normal tissues of TCGA (n=715 from 18 primary sites). The intensity of purple indicates the percentile (25th, 50th, 75th, and 90th) of the FPKM value of each HAMP in a given tissue type. **b** Heatmap shows the mRNA expression levels of the HAMP genes across TCGA tumors (n=10,201, with samples of 33 cancer types from 27 primary sites). The intensity of purple indicates the percentile (25th, 50th, 75th, and 90th) of the FPKM value of each HAMP in a given cancer type. **c** Heatmap shows the mRNA expression levels of the HAMP genes across large-collection of human cancer cell lines (n=2,012, with samples of 30 cancer types). The intensity of purple indicates the percentile (25th, 50th, 75th, and 90th) of the FPKM value of each HAMP in a given cancer type. The phylogenetic trees were generated by multiple sequence alignments of the full-length sequences of the proteins.

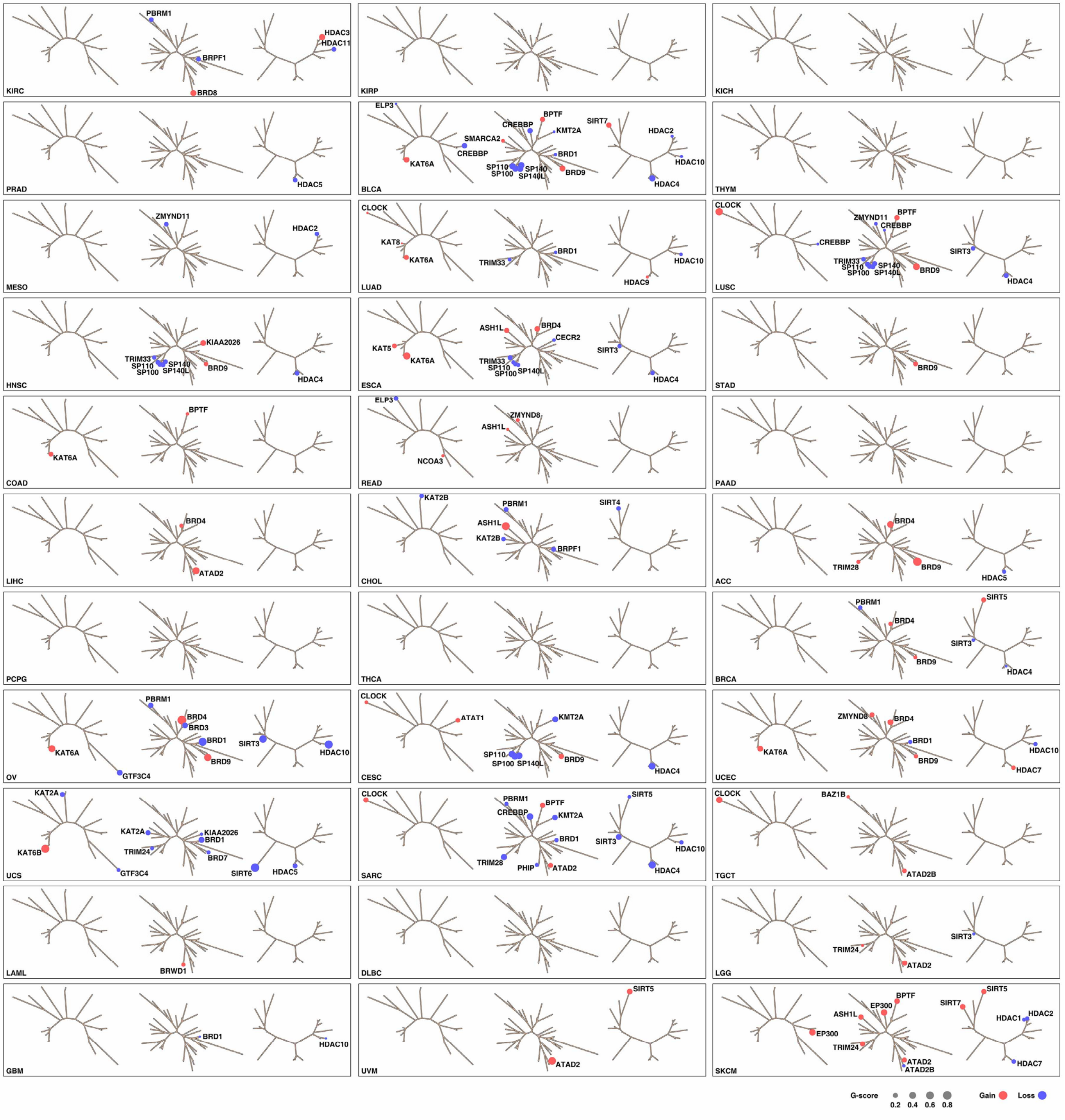

Supplementary Figure 2. Somatic copy number alterations of HAMPs in each cancer type

The bubble plot shows the G-scores, which consider both the amplitude of the aberration and the frequency of its occurrence across samples, of the putative cancer-causing HAMPs driven by SCNAs in each cancer type. The size of the bubble: G-score; red: gain; blue: loss. The phylogenetic trees were generated by multiple sequence alignments of the full-length sequences of the proteins.

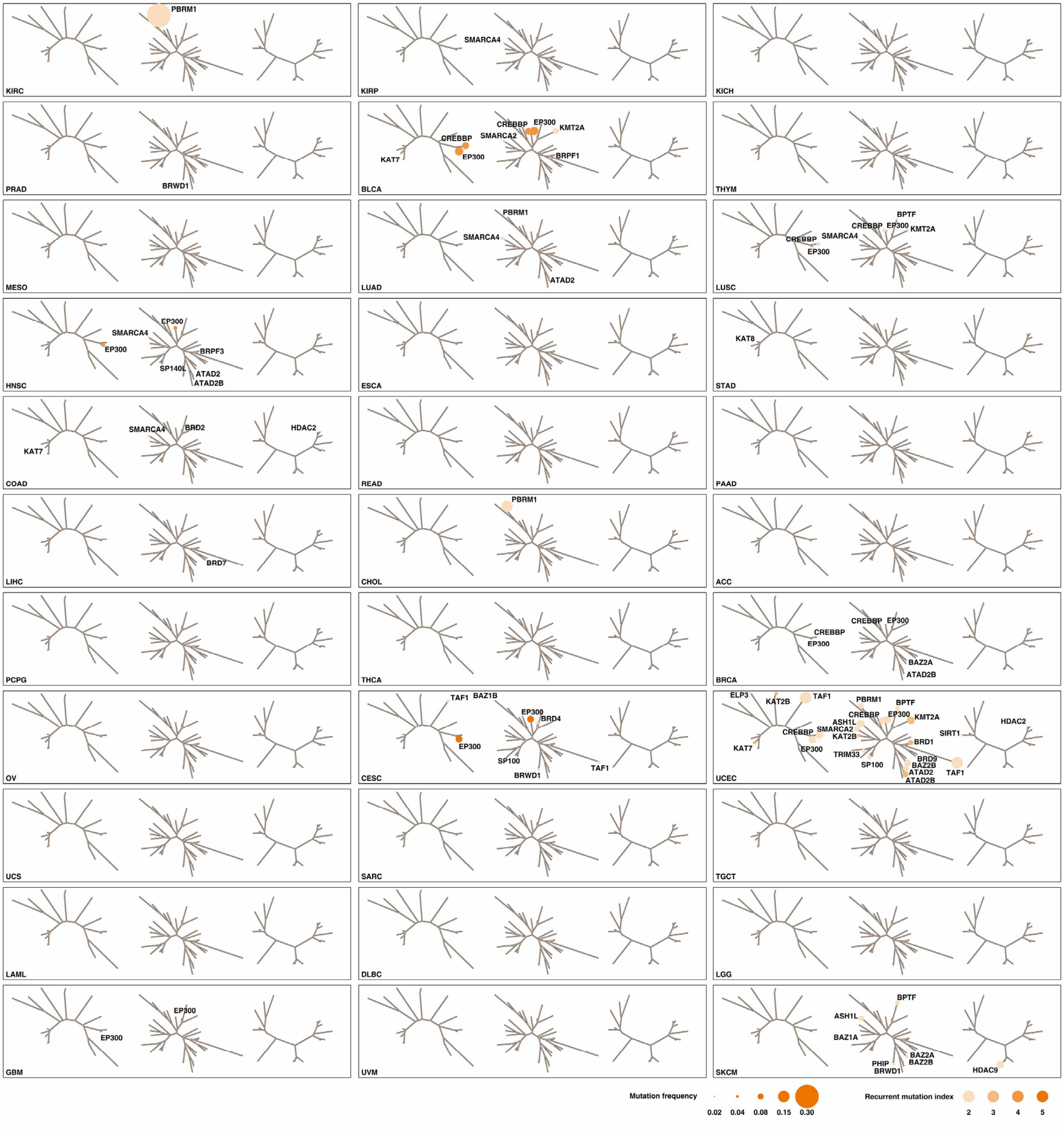

Supplementary Figure 3. Somatic mutations of HAMPs in each cancer type

The bubble plot shows the mutation frequencies and mutation indexes of the putative cancer-causing HAMPs driven by somatic mutations in each cancer type. The size of the bubble: mutation frequency; intensity of color: mutation index. The phylogenetic trees were generated by multiple sequence alignments of the full-length sequences of the proteins.

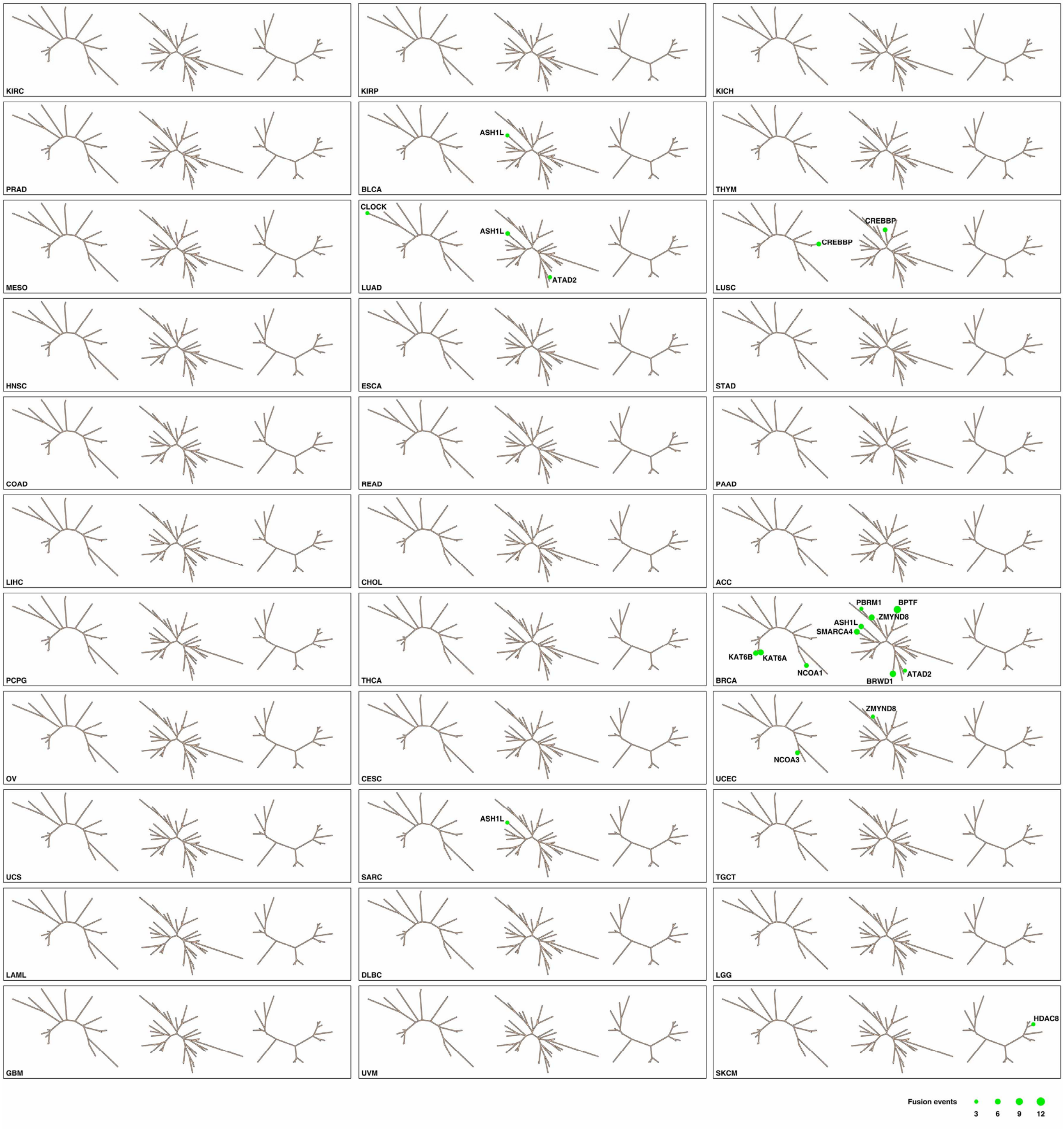

Supplementary Figure 4. Transcript fusion of HAMPs in each cancer type

The bubble plot shows the numbers of transcript fusions of each HAMP in an individual cancer type. The size of the bubble indicates the number of transcript fusions. The phylogenetic trees were generated by multiple sequence alignments of the full-length sequences of the proteins.

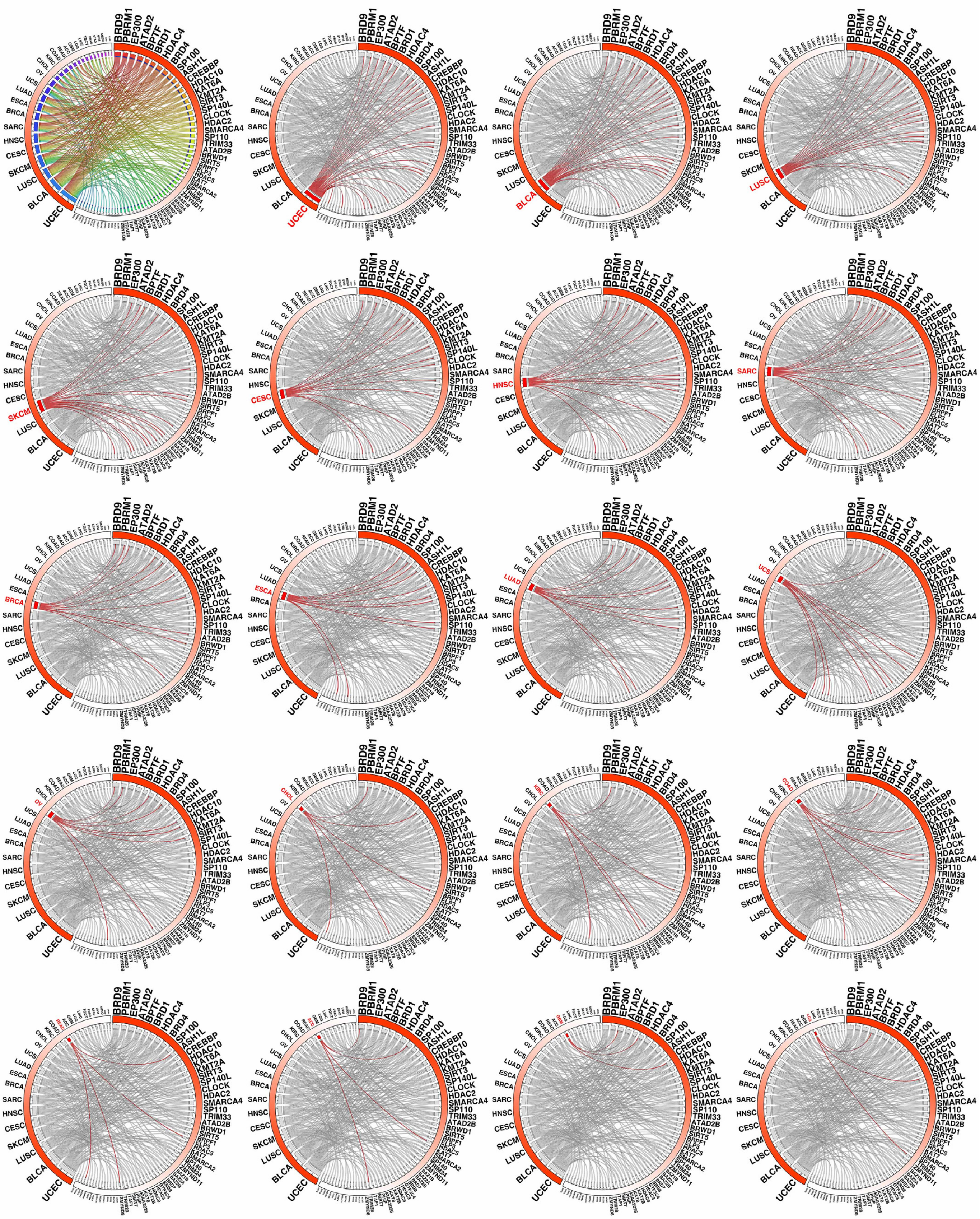

BRD9
PBRM1
EP300
ATAD2
BPTF
BRD1
HDAC4
BRD4
SP100
ASH1L
CREBBP
HDAC10
KAT6A
KMT2A
SIRT3
SP140L
CLOCK
HDAC2
SMARCA4
SP110
TRIM33
ATAD2B
BRWD1
SIRT5
UCEC
BLCA
LUSC
SKCM
CESC
HNSC
SARC
BRCA
ESCA
LUAD
UCS
OV
CHOL
KIRC
COAD

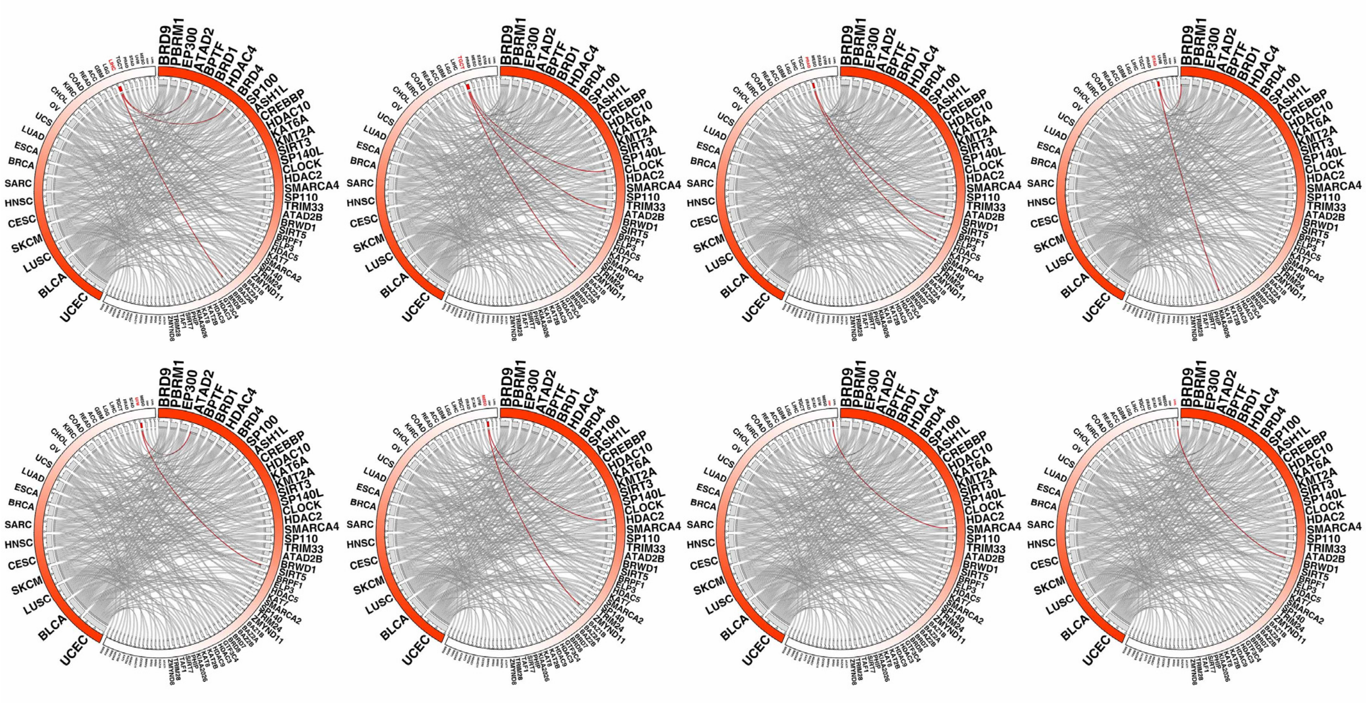

Supplementary Figure 5. The cancer types that harbor the putative cancer-causing HAMPs
The associations between a given cancer types (red) and the associated putative cancer-causing HAMPs are indicated by red lines. HAMPs were ranked by cancer recurrent score and the cancer types were ranked by the numbers of the putative cancer-causing HAMPs identified.

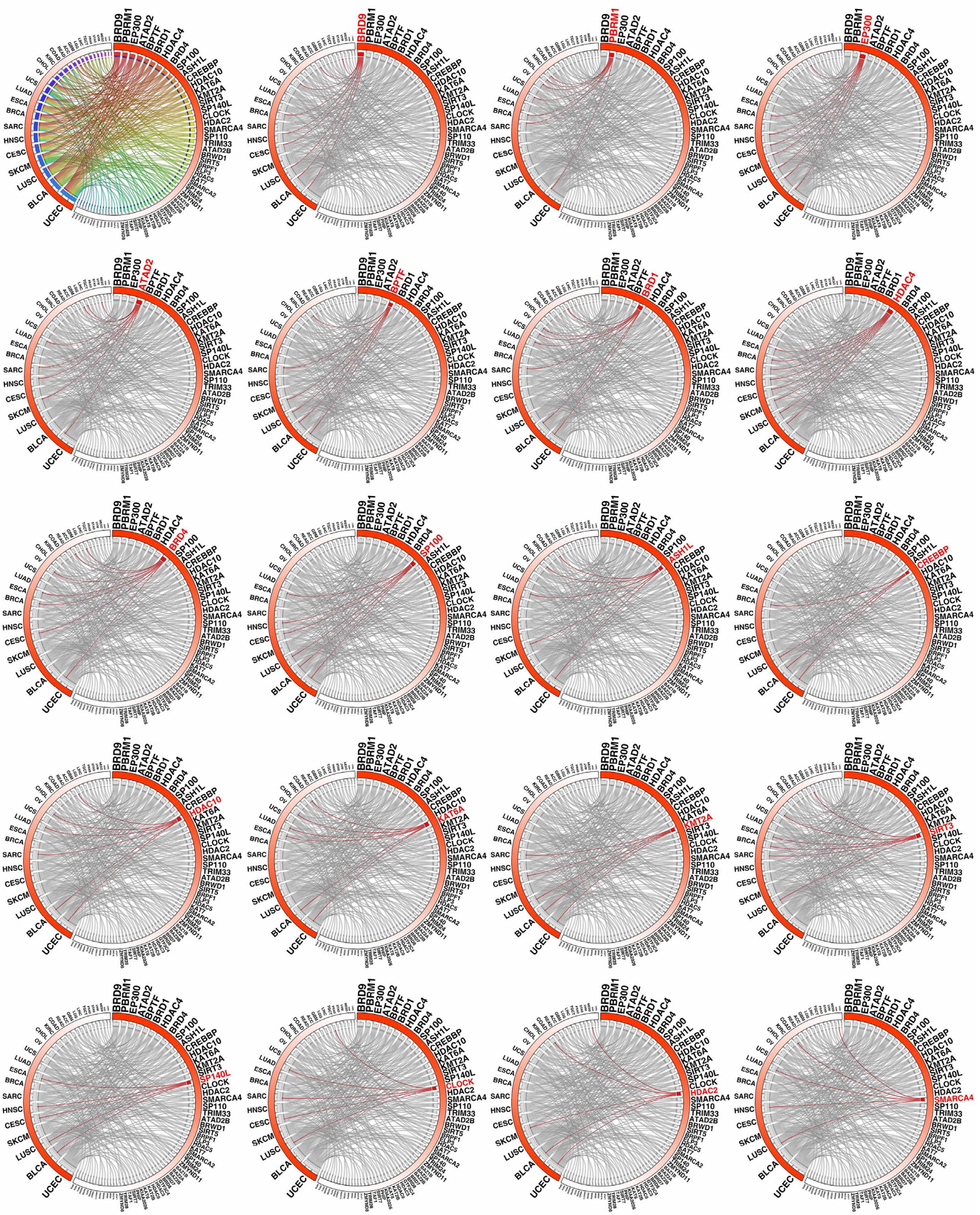
BRD9
PBRM1
EP300
ATAD2
BPTF
BRD1
HDAC4
BRD4
SP100
ASH1L
CREBBP
HDAC10
KAT6A
KMT2A
SIRT3
SP140L
CLOCK
HDAC2
SMARCA4
SP110
TRIM33
ATAD2B
BRWD1
SIRT5
UCEC
BLCA
LUSC
SKCM
CESC
HNSC
SARC
BRCA
ESCA
LUAD
UCS
OV
CHOL

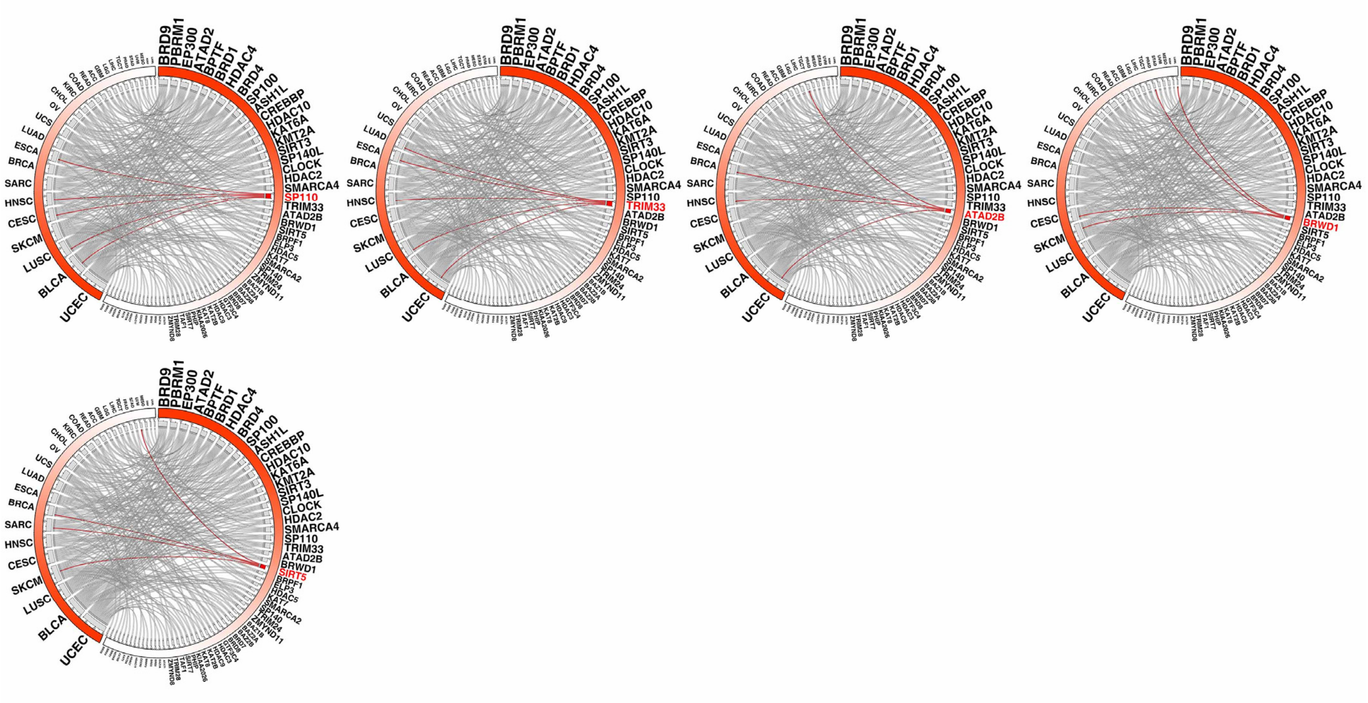

Supplementary Figure 6. The putative cancer-causing HAMPs in each cancer types
The associations between a given putative cancer-causing HAMP (red) and the cancer types harboring its genomic alteration are indicated by red lines. HAMPs were ranked by cancer recurrent score and the cancer types were ranked by the numbers of the putative cancer-causing HAMPs identified.

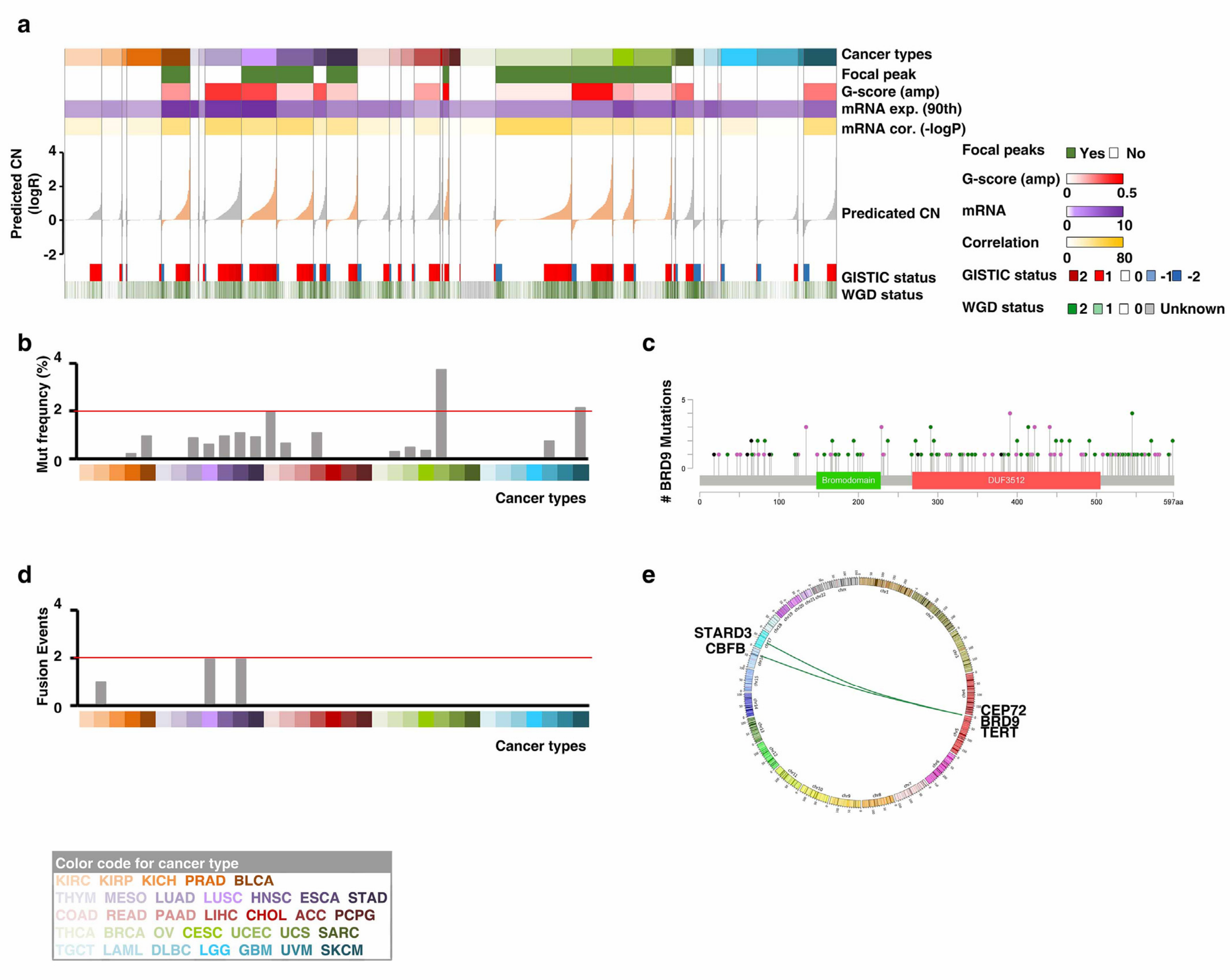

Supplementary Figure 7. Genomic alterations of BRD9 gene across 33 cancer types

a Overview of BRD9 SCNAs across 33 common adult cancers. The cancer type is color-coded and labeled on the top of the figure. The upper four panels show focal peak, G-score, the 90th percentile of mRNA expression (FPKM), and the correlation between mRNA expression and predicted copy number (-log[p-value]) for each TCGA cancer type. The middle panel shows the predicted BRD9 copy numbers of each patient across 33 cancer types. In each cancer type, the patients are ordered by predicted BRD9 copy numbers. The cancer types with BRD9 recurrent amplification are marked as yellow. The lower two panels show the GISTIC status and whole genome doubling (WGD) status of each patient. **b** Frequency of BRD9 mutations across 33 common adult cancers. Note: in most cancer types, BRD9 gene has low mutation frequency (average 0.74% across all TCGA specimens). Only in UCEC and SKCM (both are high mutation burden cancers) that BRD9 gene has relatively high mutation frequencies (>2%). **c** The lollipop plot illustrates the distribution and categories of somatic mutations in the BRD9 gene-coding sequences across all cancer types. The mutations are randomly distributed along the entire coding sequence, and no hotspot mutation was identified. **d** Numbers of BRD9 transcript fusions across 33 common adult cancers. Note: only five transcript fusion events were identified in three cancer types (ESCA, KIRP, and LUSC). **e** The fusions of BRD9 in TCGA samples are shown in Circos plots. The fused genes are illustrated as lines that connect two parental genes.

Supplementary Methods

Gene annotation

The GENCODE comprehensive gene annotation version 22[1,2] was downloaded from the GENCODE website (https://www.gencodegenes.org/releases/22.html). It was used to define the gene features including chromosome position, transcript structure, as well as transcript and protein sequences. The human reference genome GRCh38/hg38 was downloaded from the UCSC Genome Browser website (http://hgdownload.cse.ucsc.edu/goldenPath/hg38/bigZips/). It was used as the genome assembly.

Phylogenetic trees of the HAMP protein families

The full-length amino acid sequences of HAMP proteins were aligned using multiple alignment tool ClustalW (http://www.clustal.org/). The resulted sequence alignments were used to construct phylogenetic trees by MEGA7[3] with the maximum likelihood evolution algorithm. A Poisson correction was used for multiple substitution models. Initial trees for the heuristic search were obtained automatically by applying Neighbor-Join (NJ) and Bio-NJ algorithms to a matrix of pairwise distances estimated using a Jones-Taylor-Thornton (JTT) model, and then selecting the topology with superior log likelihood value[3]. If a protein contains multiple acetylation associated domains among the writer, reader, and eraser groups, it was presented in all corresponding trees. E.g., EP300 contains both HAT and BRD domains, thus, it was showed in both writer and reader phylogenetic trees.

t-SNE analysis

The correlation between HAMP gene expression profiles and cancer types was visualized by t-Distributed Stochastic Neighbor Embedding (t-SNE) analysis developed by van der Maaten and Hinton[4] (https://lvdmaaten.github.io/tsne/) .

Database search for publications, patent applications and clinical trials

Data search was performed to identify the HAMP genes that were involved in published research works, patent applications and clinical trials. Three open access databases were surveyed: (1) The PubTator/NCBI (https://www.ncbi.nlm.nih.gov/CBBresearch/Lu/Demo/PubTator/), a web-based text mining tool for assisting biocuration[5], was used to retrieve the publications related to the HAMP genes. The PubTator score retrieved from the Target Central Resource Database was used to define the under-studied HAMP gene (i.e., PubTator score < 150). (2) The Patentscope, a patent database of Intellectual Property Organization (http://www.wipo.int/patentscope/en/), was used to retrieve the patents related to the HAMP genes. (3) The ClinicalTrials.gov (https://www.clinicaltrials.gov/) databased was used to retrieve the clinical trials related to the HAMP genes.

References

1 Djebali, S. *et al.* Landscape of transcription in human cells. *Nature* **489**, 101-108, doi:10.1038/nature11233 (2012).

2 Harrow, J. *et al.* GENCODE: the reference human genome annotation for The ENCODE Project. *Genome Res* **22**, 1760-1774, doi:10.1101/gr.135350.111 (2012).

3 Kumar, S., Stecher, G. & Tamura, K. MEGA7: Molecular Evolutionary Genetics Analysis Version 7.0 for Bigger Datasets. *Mol Biol Evol* **33**, 1870-1874, doi:10.1093/molbev/msw054 (2016).

4 van der Maaten, L., Hinton, G. Visualizing Data using t-SNE. *Journal of Machine Learning Research* 2579-2605 (2008).

5 Wei, C. H., Kao, H. Y. & Lu, Z. PubTator: a web-based text mining tool for assisting biocuration. *Nucleic Acids Res* **41**, W518-522, doi:10.1093/nar/gkt441 (2013).
