# Supplementary material for: Genomic characterization of genes encoding histone acetylation modulator proteins identifies therapeutic targets for cancer treatment
Source: Nat Commun. 2019 Feb 13;10:733. doi: 10.1038/s41467-019-08554-x (PMC6374416; doi:10.1038/s41467-019-08554-x)
Supplement: Supplementary file 2 — Description of Additional Supplementary Files [file 41467_2019_8554_MOESM2_ESM.docx]

**Description of Additional Supplementary Files**

File Name: Supplementary Data 1

Description: List of the HAMPs in the human genome.

File Name: Supplementary Data 2

Description: Summary of TCGA specimens.

File Name: Supplementary Data 3

Description: Summary of HAMP mRNA expression levels (FPKM value) in each cancer type.

File Name: Supplementary Data 4

Description: Summary of the HAMPs located in the recurrent focal SCNAs identified by GISTIC in each cancer type.

File Name: Supplementary Data 5

Description: Summary of the G-score of the HAMPs in each cancer type.

File Name: Supplementary Data 6

Description: List of the putative cancer-causing HAMPs driven by SCNAs in each cancer type.

File Name: Supplementary Data 7

Description: Summary of the co-altered genes located in the identified recurrent focal SCNAs in each cancer type.

File Name: Supplementary Data 8

Description: Pan-cancer level overall G-score of HAMPs that recurrently gained or lost copy number in at least one cancer types.

File Name: Supplementary Data 9

Description: Summary of the M-score of the HAMPs in each cancer type.

File Name: Supplementary Data 10

Description: List of the putative cancer-causing HAMPs driven by somatic mutations in each cancer type.

File Name: Supplementary Data 11

Description: Summary of mutation frequency of the HAMPs in each cancer type.

File Name: Supplementary Data 12

Description: Pan-cancer level overall M-score of the HAMPs.

File Name: Supplementary Data 13

Description: Summary of the mutation categories of the HAMPs in each cancer type.

File Name: Supplementary Data 14

Description: Summary of the mutation types of the HAMPs in each cancer type.

File Name: Supplementary Data 15

Description: Summary of the mutation timing status of the HAMPs in each cancer type.

File Name: Supplementary Data 16

Description: Summary of the mutation clonal heterogeneity of the HAMPs in each cancer type.

File Name: Supplementary Data 17

Description: Summary of the mutations within the significantly mutated HAMP Pfam domains.

File Name: Supplementary Data 18

Description: List of the transcript fusions of the HAMPs in each cancer type.

File Name: Supplementary Data 19

Description: Summary of the transcript fusions of the HAMPs in each cancer type.

File Name: Supplementary Data 20

Description: Summary of the overall recurrent scores of the HAMPs across common cancer types.

File Name: Supplementary Data 21

Description: Summary of the total genomic alteration events of the HAMPs in each cancer type.

File Name: Supplementary Data 22

Description: Summary of the PubTator score, patent applications, targeting small molecules, and related clinical trials for the HAMPs.

File Name: Supplementary Data 23

Description: List of the clinical trials with the HAMPs listed as primary targets.

File Name: Supplementary Data 24

Description: List of the BRD9-associated molecular pathways identified by Guilt-by-Association (GBA) analysis.
