# Supplementary material for: Genomic characterization of genes encoding histone acetylation modulator proteins identifies therapeutic targets for cancer treatment
Source: Nat Commun. 2019 Feb 13;10:733. doi: 10.1038/s41467-019-08554-x (PMC6374416; doi:10.1038/s41467-019-08554-x)
Supplement: Supplementary file 28 — Reporting Summary [file 41467_2019_8554_MOESM28_ESM.pdf]

nature research

Corresponding author(s): Lin Zhang

Reporting Summary

Nature Research wishes to improve the reproducibility of the work that we publish. This form provides structure for consistency and transparency in reporting. For further information on Nature Research policies, see Authors & Referees and the Editorial Policy Checklist.

Statistical parameters

When statistical analyses are reported, confirm that the following items are present in the relevant location (e.g. figure legend, table legend, main text, or Methods section).

n/a	Confirmed	
☐	☒	The exact sample size (n) for each experimental group/condition, given as a discrete number and unit of measurement
☐	☒	An indication of whether measurements were taken from distinct samples or whether the same sample was measured repeatedly
☐	☒	The statistical test(s) used AND whether they are one- or two-sided<br>*Only common tests should be described solely by name; describe more complex techniques in the Methods section.*
☐	☒	A description of all covariates tested
☐	☒	A description of any assumptions or corrections, such as tests of normality and adjustment for multiple comparisons
☐	☒	A full description of the statistics including central tendency (e.g. means) or other basic estimates (e.g. regression coefficient) AND variation (e.g. standard deviation) or associated estimates of uncertainty (e.g. confidence intervals)
☐	☒	For null hypothesis testing, the test statistic (e.g. F, t, r) with confidence intervals, effect sizes, degrees of freedom and P value noted<br>*Give P values as exact values whenever suitable.*
☒	☐	For Bayesian analysis, information on the choice of priors and Markov chain Monte Carlo settings
☒	☐	For hierarchical and complex designs, identification of the appropriate level for tests and full reporting of outcomes
☒	☐	Estimates of effect sizes (e.g. Cohen's d, Pearson's r), indicating how they were calculated
☐	☒	Clearly defined error bars<br>*State explicitly what error bars represent (e.g. SD, SE, CI)*

Our web collection on statistics for biologists may be useful.

Software and code

Policy information about availability of computer code

Data collection	The gene level RNA expression data of TCGA were downloaded from the GDC Data Portal (https://portal.gdc.cancer.gov/) (retrieved date: Oct, 27, 2017).<br><br>The single-nucleotide polymorphism array data (Affymetrix Genome-Wide Human SNP Array 6.0) in CEL format of TCGA were downloaded from the TCGA Data Portal (https://tcga-data.nci.nih.gov/tcga/).<br><br>Segmentation files of TCGA tumor samples processed by circular binary segmentation (CBS) algorithm were retrieved from the GDAC Firehose of the Broad Institute (http://gdac.broadinstitute.org/) (retrieved date: Jan, 3, 2018).<br><br>Mutation Annotation Format (MAF) profiles for 33 cancer types were downloaded from the TCGA Multi-Center Mutation Calling in Multiple Cancers (MC3) project (https://doi.org/10.7303/syn7214402) (retrieved date: Nov, 6, 2017).<br><br>The gene fusion data of TCGA were retrieved from TumorFusions data portal (http://tumorfusions.org/) (retrieved date: Jan, 16, 2018).
Data analysis	GISTIC v2.0.23 (https://www.broadinstitute.org/cancer/cga/gistic) was used to identify significantly recurrent focal genomic regions that were gained or lost in a given tumor type.<br><br>HAPSEG v1.1.1 (http://software.broadinstitute.org/cancer/software/genepattern/modules/docs/HAPSEG/1) was used to generate copy number data segmented by haplotype.

ABSOLUTE v1.0.6 (http://archive.broadinstitute.org/cancer/cga/absolute) was used to estimate intra-tumor heterogeneity.

MutSigCV v1.4 (http://software.broadinstitute.org/cancer/software/genepattern/modules/docs/MutSigCV), Oncodrivefm v1.0.1 (http://bg.upf.edu/group/projects/oncodrive-fm.php), OncodriveCLUST v1.0.0 (http://bg.upf.edu/group/projects/oncodrive-clust.php), ActiveDriver v0.0.10 (http://reimandlab.org/software/activedriver/), and HotSpot3D v1.8.1 (https://github.com/ding-lab/hotspot3d) were used to predict the putative cancer-causing HAMP genes driven by mutation.

LowMACA v1.12.0 (https://www.bioconductor.org/packages/release/bioc/html/LowMACA.html) was used to analyze the mutation profile of multiple proteins via consensus alignment and identify their mutational hotspots within Pfam domains.

For manuscripts utilizing custom algorithms or software that are central to the research but not yet described in published literature, software must be made available to editors/reviewers upon request. We strongly encourage code deposition in a community repository (e.g. GitHub). See the Nature Research guidelines for submitting code & software for further information.

Data

Policy information about availability of data

All manuscripts must include a data availability statement. This statement should provide the following information, where applicable:

- Accession codes, unique identifiers, or web links for publicly available datasets
- A list of figures that have associated raw data
- A description of any restrictions on data availability

This study is based upon data generated by The Cancer Genome Atlas project established by the NCI and NHGRI. Information about TCGA and the investigators and institutions that constitute the TCGA research network can be found at http://cancergenome.nih.gov. All relevant data used for the current study are available through the Genomic Data Commons portal (https://gdc-portal.nci.nih.gov), the TCGA Data Portal (https://tcga-data.nci.nih.gov/tcga/), the GDAC Firehose of the Broad Institute (http://gdac.broadinstitute.org/), the TCGA Multi-Center Mutation Calling in Multiple Cancers (MC3) project (https://doi.org/10.7303/syn7214402), and TumorFusions data portal (http://tumorfusions.org/).

Field-specific reporting

Please select the best fit for your research. If you are not sure, read the appropriate sections before making your selection.

☒ Life sciences ☐ Behavioural & social sciences ☐ Ecological, evolutionary & environmental sciences

For a reference copy of the document with all sections, see nature.com/authors/policies/ReportingSummary-flat.pdf

Life sciences study design

All studies must disclose on these points even when the disclosure is negative.

Sample size

No statistical methods were used to predetermine sample size. Sample size was determined by the number of cases available in the databases mined. The TCGA database used for the current study were composed of RNA-Seq data (10,201 tumor specimens and 730 corresponding normal adjacent specimens), SNP array data (10,950 specimens), Exome-seq data (10,224 specimens), and transcript fusion data (9,799 specimens).

Data exclusions

No sample or animal was excluded from the analysis for in vitro and in vivo experiments in Figure 8.

For TCGA analysis, If more than one profiling file (sample) existed for a patient in TCGA, one single file will be selected and used in analysis based on the following rules:

For RNA-seq analysis: If more than one sample existed for a participant, one single tumor sample (and matched adjacent sample, if applicable) was selected based on the following rules: (1) tumor sample type: primary (01) > recurrent (02) > metastatic (06); (2) order of sample portions: higher portion numbers were selected; and (3) order of plate: higher plate numbers were selected.

For SNP array analysis: Sample selection based on following rules: (1) sample type: for tumor tissues, primary (01) > recurrent (02) > metastatic (06); for normal control tissues, blood (10) > solid (11); (2) molecular type of analyte for analysis: prefer D analytes (native DNA) over G, W, or X (whole-genome amplified); (3) order of sample portions: higher portion numbers were selected; and (4) order of plate: higher plate numbers were selected.

For WES analysis: If multiple samples existed for a participant in the MAF, one single pair of tumor/matched control sample was kept following the rules: (1) sample type: for tumor tissues, primary (01) > recurrent (02) > metastatic (06); for normal tissues, blood (10) > solid (11); (2) molecular type of analyte for analysis: prefer D analytes (native DNA) over G, W, or X (whole-genome amplified); (3) order of sample portions: higher portion numbers were selected; and (4) order of plate: higher plate numbers were selected. We excluded all mutations that were not tagged with “PASS” or “WGA” alone in all cancer types.

For transcript fusion analysis: If more than one sample existed for a participant, one single sample was kept following the rules: (1) sample type: for tumor tissues, primary (01) > recurrent (02) > metastatic (06); (2) order of sample portions: higher portion numbers were selected; and (3) order of plate: higher plate numbers were selected.

Replication

We have provided the complete analysis methods as Methods for reproducibility.

Replication	Biophysical measurements (Figure 8) were repeated with different samples every time in technical triplicate.<br>The genomic analysis was only performed in TCGA cohort (no replication sample cohort is available for TCGA).
Randomization	Conventional randomization process was not relevant to this study. However, the animals were randomized into two groups before tumor cell injection.
Blinding	Blinding was not relevant to this study. Due to the nature of the performed experiments, no blinding was used as it was deemed unfeasible. However, the resulting tumors were analyzed in a blinded manner.

Reporting for specific materials, systems and methods

Materials & experimental systems

n/a	Involved in the study	
☒	☐	Unique biological materials
☒	☐	Antibodies
☐	☒	Eukaryotic cell lines
☒	☐	Palaeontology
☐	☒	Animals and other organisms
☒	☐	Human research participants

Methods

n/a	Involved in the study	
☒	☐	ChIP-seq
☒	☐	Flow cytometry
☒	☐	MRI-based neuroimaging

Eukaryotic cell lines

Policy information about cell lines

Cell line source(s)	Cancer cell lines MDA-MB-231, HCC1937, OVCAR8, and Caov3 were purchased from ATCC or NCI Development Therapeutics Program.
Authentication	Cancer cell lines were purchased from the ATCC and NCI Development Therapeutics Program without further authentication.
Mycoplasma contamination	Cells were tested for mycoplasma contamination using Mycoplasma Plus PCR Primer Set (Agilent, Santa Clara, CA) and confirmed that they are mycoplasma negative.
Commonly misidentified lines<br>(See ICLAC register)	No commonly misidentified cell lines were used in this study.

Animals and other organisms

Policy information about studies involving animals; ARRIVE guidelines recommended for reporting animal research

Laboratory animals	6-8 weeks female nude mice were purchased from Jackson Laboratories. All research involving animals was complied with protocols approved by the University of Pennsylvania Animal Care and Use Committee.
Wild animals	The study did not involve wild animals.
Field-collected samples	The study did not involve samples collected from the field.
